# Cytokeratin 8 as a Novel Therapeutic Target in Type 2 Diabetes Mellitus: Suppression of Hepatic Glycogen Synthesis *via* IRS1/PI3K/Akt/GSK3β Signaling

**DOI:** 10.2174/0113894501394500250903095958

**Published:** 2025-10-01

**Authors:** Mingzhu Sun, Xiuli Li, Jin Sun, Zhidong Wang

**Affiliations:** 1 Department of Endocrinology, The Second Affiliated Hospital of Xi’an Jiaotong University, Xi’an, Shaanxi, 710004, P.R. China;; 2 National & Local Joint Engineering Research Center of Biodiagnostics and Biotherapy, The Second Affiliated Hospital of Xi’an Jiaotong University, Xi’an, Shaanxi, 710004, P.R. China;; 3 Department of General Surgery, The Second Affiliated Hospital of Xi’an Jiaotong University, Xi’an, Shaanxi, 710004, P.R. China

**Keywords:** Type 2 diabetes mellitus, hepatic glycogen synthesis, IRS1/PI3K/Akt/GSK3β pathway, cytokeratin 8, therapeutic target, insulin resistance, hepatic glucose metabolism

## Abstract

**Introduction:**

Recent studies have established that cytokeratin 8 (CK8) is closely linked to glycogen synthesis; however, its mechanistic role in hepatic glycogen synthesis in type 2 diabetes mellitus (T2DM) remains unclear. This study aimed to elucidate the effects and underlying molecular mechanisms of CK8.

**Methods:**

We analyzed CK8 expression and the IRS1 (Insulin Receptor Substrate 1)/PI3K (Phosphoinositide 3-Kinase)/Akt (Protein Kinase B)/GSK3β (Glycogen Synthase Kinase 3 beta) pathway in liver samples from T2DM patients, diabetic C57BL/6J mouse models, and high glucose-treated NCTC 1469 cells using Western blotting, immunohistochemistry, and PAS staining.

**Results:**

CK8 was significantly upregulated in all T2DM models, correlating with suppressed IRS1/PI3K/Akt/GSK3β signaling and reduced glycogen synthesis. Our functional studies demonstrated that CK8 overexpression exacerbated these effects, while CK8 knockdown restored glycogen levels to near-normal.

**Discussion:**

In our study, CK8 was identified as a negative regulator of hepatic glycogen synthesis by modulating the IRS1/PI3K/Akt/GSK3β pathway.

**Conclusion:**

These findings position CK8 as a promising therapeutic target for T2DM, with CK8 inhibition offering a novel strategy to improve hepatic insulin resistance and glycogen storage without requiring β-cell stimulation.

## 1 INTRODUCTION

Type 2 diabetes mellitus (T2DM) is a metabolic disorder and the third leading cause of global mortality [1]. Characterized by chronic hyperglycemia, T2DM contributes to multisystem complications, including renal, cardiovascular, and neurological damage [2, 3]. According to the World Health Organization, approximately 422 million individuals worldwide suffer from diabetes mellitus, with over 90% of adult cases attributed to T2DM [4, 5]. The pathogenesis of T2DM involves insulin resistance, pancreatic β-cell dysfunction, and impaired compensatory insulin secretion [6, 7]. Multiple organs, such as the liver, skeletal muscle, and adipose tissue, play critical roles in glucose dysregulation, further exacerbating the disease [8]. Although current therapies (*e.g.*, insulin and synthetic hypoglycemic agents) remain the cornerstone of clinical management, their use is often limited by adverse effects, including hypoglycemia, gastrointestinal disturbances, and allergic reactions [9, 10]. Thus, identifying novel molecular targets to restore glucose homeostasis remains an urgent priority.

Emerging evidence suggests that dysregulated insulin signaling, aberrant lipid metabolism, and chronic inflammation contribute to the progression of T2DM [11-13]. However, the precise molecular mechanisms underlying these processes remain incompletely understood. The contribution of cytoskeletal proteins to hepatic metabolic dysregulation remains underexplored, particularly their role in modulating insulin signaling cascades. Notably, the cytokeratin (CK) family, a subclass of intermediate filament proteins, has garnered attention for its role in cellular structure and metabolic regulation [14] Among these, cytokeratin 8 (CK8) is highly expressed in hepatocytes and epithelial tissues, where it modulates diverse physiological functions, including cell differentiation, apoptosis, and drug resistance [15-18]. Recent studies have suggested that CK8 may also influence hepatic glycogen synthesis; however, its precise mechanistic interplay with insulin signaling pathways in T2DM remains unresolved [19, 20].

The IRS1 (Insulin Receptor Substrate 1)/PI3K (Phosphoinositide 3-Kinase)/Akt (Protein Kinase B)/GSK3β (Glycogen Synthase Kinase 3 beta) pathway is a well-established regulator of insulin signaling and glucose metabolism [21-23]. Dysfunction of this pathway contributes to hepatic insulin resistance and impaired glycogen synthesis in type 2 diabetes mellitus (T2DM) [24-28]. Notably, preliminary data suggest that CK8 may interact with the IRS1/PI3K/Akt/GSK3β cascade, potentially linking cytoskeletal dynamics to metabolic regulation [29-32]. Despite these advances, the functional relationship between CK8, this pathway, and hepatic glycogen synthesis in T2DM has not been systematically investigated. The mechanistic role of CK8 in modulating IRS1 function and glycogen synthesis remains incompletely defined. Current evidence suggests CK8 may impair hepatic insulin signaling through multiple pathways, including direct physical interaction with IRS1, potentially promoting its degradation *via* ubiquitin-proteasome systems; dysregulation of insulin signaling component localization (*e.g.*, altered membrane trafficking of PI3K/Akt), and synergistic effects with T2DM-related stressors, such as oxidative stress, exacerbating glycogen synthesis suppression. While the suppression of GSK3β phosphorylation by CK8 is a primary mediator, contributions from other kinases (*e.g.*, AMPK (Adenosine Monophosphate-activated Protein Kinase), PKC (Protein kinase C), *etc.*) cannot be excluded. Critically, no systematic studies have validated these hypotheses, particularly regarding the specificity of CK8-IRS1 binding, the subcellular redistribution of signaling molecules, or the effects of combinatorial stressors. Elucidating these mechanisms is essential to establishing the causal role of CK8 in T2DM-associated insulin resistance.

This study aimed to elucidate the role of CK8 in modulating hepatic glycogen synthesis *via* the IRS1/PI3K/Akt/GSK3β pathway in T2DM. Using a combination of clinical samples (liver samples from T2DM patients), a murine T2D model, and an *in vitro* high-glucose NCTC (National Collection of Type Cultures) 1469 cell system, we assessed the interplay between CK8 expression and glycogen metabolic pathways. This study bridges these knowledge gaps by elucidating the role of CK8 in hepatic glycogen depletion through multisystem validation (human, murine, and cellular models). We demonstrate that the upregulation of CK8 directly correlates with the suppression of the IRS1/PI3K/Akt pathway. CK8 operates as a nodal regulator at the intersection of cytoskeletal remodeling and metabolic dysfunction, and its targeting restores glycogen storage independently of β-cell function. Our findings may provide new insights into therapeutic strategies for restoring glycogen storage in T2DM.

## 2 MATERIALS AND METHODS

### 2.1 Reagents and Antibodies

Antibodies, including anti-IRS1, anti-phosphorylated(p)-IRS1, anti-PI3K, anti-p-PI3K, anti-Akt, anti-p-Akt, anti-GSK3β, anti-p-GSK3β, anti-glycogen synthase (GS), anti-CK8, and anti-GAPDH (Glyceraldehyde-3-Phosphate Dehydrogenase), were purchased from Cell Signaling Technology, Inc. (Danvers, MA, USA). Cell culture reagents, including DMEM, horse serum (HS), penicillin, and streptomycin, were obtained from Procell Life Science and Technology Co., Ltd. (Wuhan, Hubei, China). The ELISA (Enzyme-Linked Immunosorbent Assay) kit and the Mouse Insulin ELISA Kit were procured from Beyotime Institute of Biotechnology (Shanghai, China). Chemicals, including RIPA (RadioImmunoPrecipitation Assay) lysis buffer and protease inhibitor cocktail, were acquired from MilliporeSigma (Burlington, MA, USA). The specific phosphorylation sites targeted by the antibodies, based on standard catalog specifications from Cell Signaling Technology, are as follows: p-IRS1 (Tyr612), Catalog #2381, detects IRS1 phosphorylated at Tyr612; p-PI3K (Tyr458/Tyr199), Catalog #4228, detects PI3K p85α phosphorylated at Tyr458 (human) or Tyr199 (murine); p-Akt (Ser473), Catalog #4060, detects Akt phosphorylated at Ser473; and p-GSK3β (Ser9), Catalog #5558, detects GSK3β phosphorylated at Ser9.

### 2.2 Patient Samples

Liver tissue samples were collected from 6 healthy controls and 6 patients with T2DM at The Second Affiliated Hospital of Xi’an Jiaotong University between March and October 2020. The diagnosis of T2DM was based on the 1999 WHO Diagnostic Criteria, defined as follows: (**1**) Fasting plasma glucose ≥7.0 mmol/L, 2-hour oral glucose tolerance test (OGTT) ≥11.1 mmol/L, or random plasma glucose ≥11.1 mmol/L; (**2**) HbA1c levels between 7.0% and 9.5%; (**3**) Uncomplicated T2DM, excluding comorbidities, such as hypertension, coronary heart disease, hepatitis, or other systemic conditions; (**4**) No prior use of glucose-lowering medications or treatments for T2DM and related disorders. The 6 healthy control subjects were volunteers with confirmed normal glucose metabolism based on clinical evaluation. They maintained a stable, healthy diet and had no history of diabetes, glucose-lowering medication use, or related metabolic disorders. Key demographic and metabolic parameters (including age, sex, body mass index (BMI), HbA1c levels, and fasting blood glucose) are summarized in Table **1**. All participants provided written informed consent for liver biopsy and the use of their tissues for research purposes. The study protocol was approved by the Medical Ethics Committee of Xi’an Jiaotong University Health Science Center (Approval No. 2019-197), in compliance with the Declaration of Helsinki and institutional guidelines.

### 2.3 Cell Culture and Treatment

Murine NCTC 1469 hepatocytes (Procell Life Science & Technology Co., Ltd, Wuhan, China). were cultured in Dulbecco’s Modified Eagle Medium (DMEM) supplemented with 10% horse serum (HS), 100 μg/ml streptomycin, and 100 U/ml penicillin at 37°C in a humidified 5% CO_2_ atmosphere. For the experiments, cells were grown to 70-80% confluence and then transfected for 24 hours with one of the following: an empty vector (negative control), a CK8 overexpression plasmid, scrambled shRNA (sh-NC), or CK8-targeting shRNA (sh-CK8). Following transfection, cells were exposed to high-glucose (HG) conditions (33.3 mM glucose) for 24 hours, while normal glucose (NG) controls were maintained at 5.5 mM glucose (basal DMEM concentration).

### 2.4 Establishment of Animal Models

Eighteen 8-week-old male C57BL/6J mice (Biofavor Ltd.) were acclimatized for one week under controlled conditions (22±2°C, 50±5% humidity, 12-hour light/dark cycle). All mice included in this study had an initial average body weight of 20.3 ± 0.5 g (mean ± SD). All procedures were approved by the Medical Ethics Committee of Xi’an Jiaotong University Health Science Center (Approval No. 2019-197) and conducted in compliance with ARRIVE guidelines and the U.K. Animals (Scientific Procedures) Act, 1986.

Following acclimatization, mice were randomly assigned to six experimental groups (*n*=3/group): Normal control: Standard diet for 12 weeks; DM control: High-fat diet (HFD) for 12 weeks [33]; CK8-transfected DM: HFD + weekly 1×10^7^ pfu Ad-ZsGreen-CK8 (tail vein); Empty vector DM: HFD + weekly 1×10^7^ pfu Ad-ZsGreen-vector; shRNA-CK8 DM: HFD + weekly 1×10^7^ pfu Ad-ZsGreen-shRNA-CK8; and shRNA-NC DM: HFD + weekly 1×10^7^ pfu Ad-ZsGreen-shRNA-NC.

Diabetes was induced in all DM groups *via* intraperitoneal streptozotocin (30 mg/kg, 4 consecutive days). Successful model establishment was confirmed by fasting blood glucose levels ≥11.1 mM (33). Terminal procedures were performed under tribromoethanol anesthesia (500 mg/kg).

### 2.5 Biochemical Analyses

Serum insulin levels were quantified using a mouse insulin ELISA kit (Beyotime) following the manufacturer's protocol. Blood samples (500-700 μL) were collected *via* ocular puncture, with 150-200 μL of serum obtained for analysis. Samples were diluted 10-fold and measured in triplicate at 450 nm using a Multiskan MK3 (Thermo Fisher).

### 2.6 Oral Glucose Tolerance Tests (OGTT) and Insulin Tolerance Tests (ITT)

OGTT and ITT were conducted on C57BL/6J mice following 14 weeks of high-fat diet feeding. For both tests, blood glucose levels were measured using a standard glucometer with 20 μl blood samples collected from the tail vein. For OGTT, after an overnight fast (16 hours), mice received 2.0 g/kg D-glucose *via* oral gavage. Blood glucose levels were measured at baseline (0 min) and at 15, 30, 90, and 120 minutes post-administration. For ITT, following a 4-hour fast, mice were intraperitoneally injected with 0.75 U/kg insulin (Humulin R). Glucose levels were assessed at 0, 15, 30, 45, and 60 minutes after insulin administration.

## 3 HISTOLOGICAL PROCEDURES

### 3.1 Immunohistochemistry

Paraffin-embedded liver sections (4 μm) were processed through standard dewaxing and rehydration steps. After antigen retrieval and blocking, slides were incubated with anti-GS antibody (4°C, 60 min), followed by Dako REAL EnVision Detection (30 min, RT (room temperature)) and hematoxylin counterstaining. For cellular studies, NCTC 1469 cells (3×10^4^ cells/coverslip) were fixed (4% PFA(Paraformaldehyde)), permeabilized (0.5% Triton X-100), and processed similarly.

### 3.2 PAS (Periodic Acid-Schiff) Staining

Liver sections and fixed hepatocytes were stained using standard PAS protocol to assess glycogen content. All imaging was performed using an Olympus BX53 microscope.

### 3.3 Molecular Biology

Plasmid Construction: The murine CK8 coding sequence was amplified using the following primers: Forward: 5'-CTAGCTAGCCACCATGTCCATCAGGGTGACTCAGAA- AT-3'; Reverse: 5'-CCCAAGCTTTCACTTGGACACGACATCAGAAGACTCG-3'. CK8 shRNA (5'-CCAUGUAC- CAGAUUAAGUA-3') and scrambled control (5'-UUCUC- CGAACGUGUCACGU-3') were cloned into pDC316-ZsGreen vectors using appropriate linker sequences.

### 3.4 Western Blotting

Protein extracts from tissues or cells were separated by SDS-PAGE (Sodium Dodecyl Sulfate-Polyacrylamide Gel Electrophoresis) and transferred to nitrocellulose membranes [34-36]. After blocking (5% non-fat milk), membranes were probed with primary antibodies against IRS1 (1:2000), PI3K (1:2000), Akt (1:2000), GSK3β (1:3000), CK8 (1:2000), and GAPDH (1:4000), followed by HRP (Horseradish Peroxidase)-conjugated secondary antibodies (1:2000). Detection was performed using Enhanced Chemiluminescence (ECL). Prior studies using identical Ad-ZsGreen vectors (30) reported hepatocyte transfection efficiencies exceeding 80% *via* tail vein injection, which corroborates our methodology.

### 3.5 Statistical Analysis

Data are presented as mean ± SD (Standard Deviation). Analyses were performed using SPSS (Statistical Package for the Social Sciences) with GraphPad Prism 7.0 for visualization. Non-parametric tests were employed: the Mann-Whitney U test for two-group comparisons and the Kruskal-Wallis test with Dunn's post-hoc test for multiple comparisons. Exact *p*-values are archived and available upon request.

## 4 RESULTS

### 4.1 Upregulation of CK8 Expression and Glycogen Depletion in T2DM Liver Tissues

**Fig. (1):**
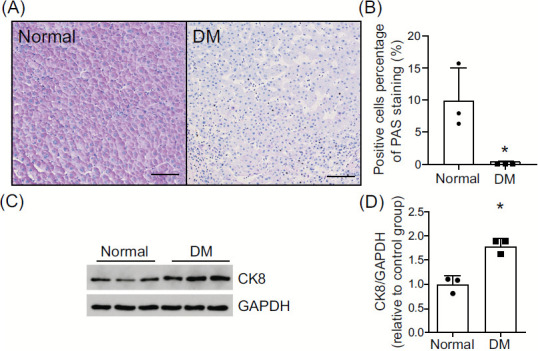
Hepatic CK8 expression and glycogen synthesis in T2DM patients vs. controls. (**A**) PAS staining of liver tissues (6 T2DM patients and 6 age-/sex-matched controls, clinical details in Table **1**). (**B**) Quantification of glycogen content (mean ± SEM, normalized to control). (**C**) Western blot of CK8 (50 μg protein/lane, GAPDH loading control). (**D**) Densitometric analysis. *p<0.05. (*n*=6/group, Mann-Whitney U test). Scale bars: 50 μm.

Glycogen content was evaluated in the liver tissues of patients with T2DM and healthy individuals *via* PAS staining. Hepatic glycogen content was significantly reduced in liver tissues from T2DM patients compared to healthy controls (Fig. **1A** and **B**). The expression of CK8 protein was subsequently detected in liver tissue *via* Western blotting. Concurrently, CK8 expression was markedly upregulated in the livers of diabetic individuals (Fig. **1C** and **D**), suggesting an inverse correlation between CK8 levels and glycogen storage. These findings indicate that CK8 overexpression may contribute to impaired glycogen synthesis in T2DM.

### 4.2 Suppression of IRS1/PI3K/Akt/GSK3β Signaling and Glycogen Synthase in Livers of Patients with T2DM

**Fig. (2):**
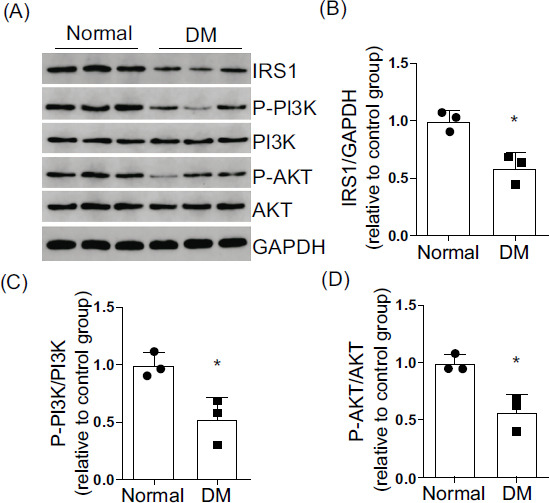
IRS1/PI3K/Akt pathway suppression in T2DM livers. (**A**) Representative blots (antibody dilutions: IRS1 1:2000, p-PI3K 1:1000). (**B-D**) Quantification of (**B**) IRS1, (**C**) p-PI3K (Tyr458), and (**D**) p-Akt (Ser473) (*n*=6/group, three technical replicates per sample). All phospho-proteins were normalized to total protein levels. (*n*=6/group, Mann-Whitney U test). *p<0.05. Scale bars: 50 μm.

**Fig. (3):**
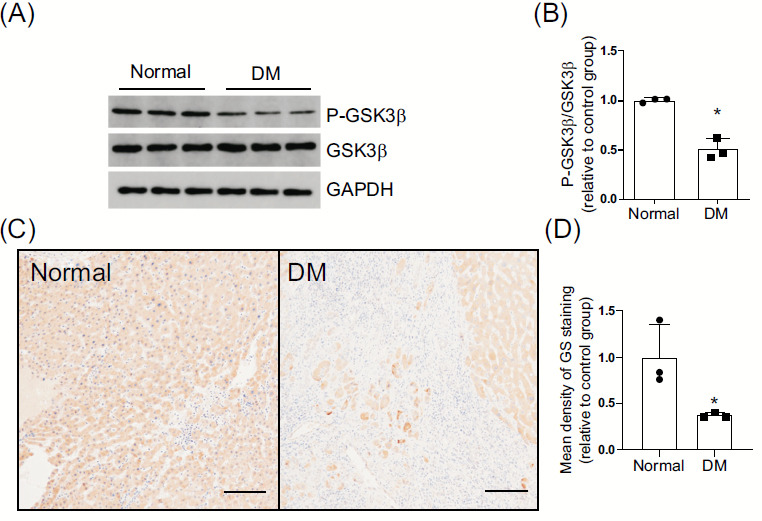
GSK3β and glycogen synthase expression in T2DM and control liver tissues. (**A**) Western blot analysis of GSK3β and p-GSK3β expression (*n*=3 per group) with GAPDH loading control. (**B**) Quantification of p-GSK3β levels. (**C**) Representative immunohistochemical staining of glycogen synthase (GS) in liver tissues. (**D**) Quantitative analysis of GS expression. *p<0.05. (*n*=6/group, Mann-Whitney U test). Scale bars: 50 μm. p-, phosphorylated; GS, glycogen synthase; T2DM, type 2 diabetes mellitus.

Western blot analysis was performed to evaluate IRS1 protein expression levels in liver tissues from patients with T2DM and healthy controls. As shown in Fig. (**2A** and **B**), IRS1 expression was significantly downregulated in T2DM patients compared to healthy individuals. Furthermore, we assessed the protein expression and phosphorylation status of PI3K and Akt by Western blotting. The results demonstrated that phosphorylation of both PI3K and Akt was markedly reduced in T2DM liver tissues, whereas their total protein levels remained unchanged (Fig. **2A**, **C**, and **D**). Additionally, we analyzed GSK3β expression, its phosphorylation state, and GS (glycogen synthase) protein levels using Western blotting and immunohistochemical staining. Compared with healthy controls, T2DM patients exhibited significantly reduced GSK3β phosphorylation, while total GSK3β protein levels showed no significant difference (Fig. **3A** and **B**) and decreased GS expression (Fig. **3C** and **D**). Collectively, these findings suggest that dysregulation of the IRS1/PI3K/Akt/GSK3β signaling pathway may contribute to impaired hepatic glycogen synthesis in T2DM.

### 4.3 High Glucose Modulates CK8 and Insulin Signaling in Hepatocytes

**Fig. (4):**
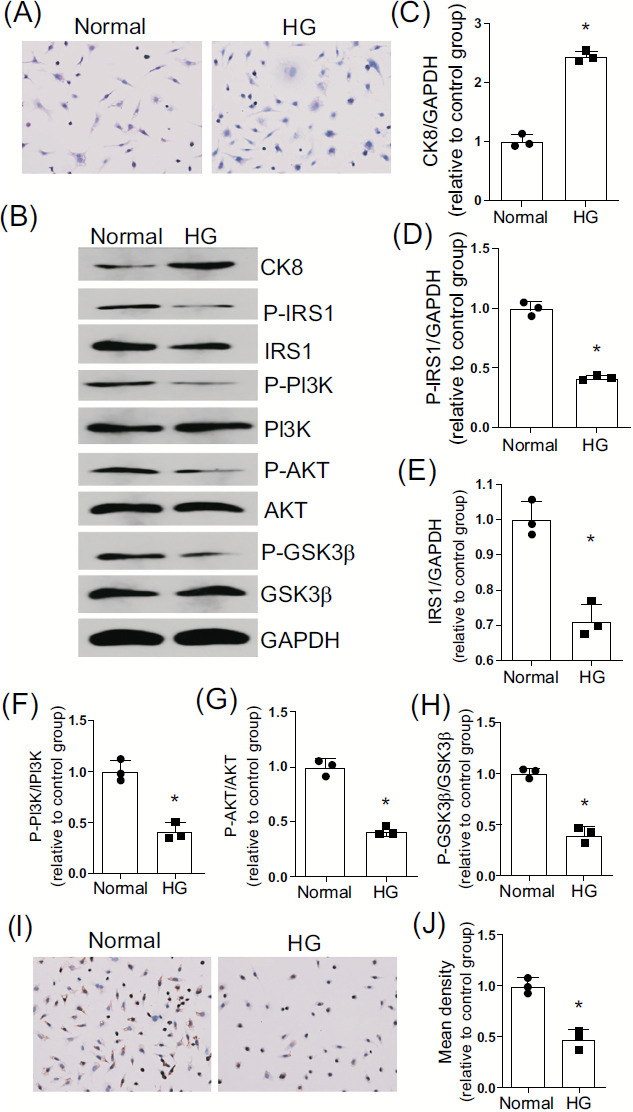
High glucose effects on glycogen metabolism in NCTC1469 cells. NCTC1469 cells treated with normal (5.5 mM) or high glucose (33.3 mM) for 24 h. (**A**) Glycogen content by PAS staining. (**B**) Western blots of CK8 and IRS1/PI3K/Akt/GSK3β pathway components (GAPDH loading control). (**C-H**) Quantification of (**C**) CK8, (**D**) p-IRS1, (**E**) IRS1, (**F**) p-PI3K, (**G**) p-Akt, and (**H**) p-GSK3β. (**I**) GS immunohistochemistry and (**J**) quantification. *p<0.05. (*n*=3 biological replicates; Kruskal-Wallis with Dunn’s post-hoc). Scale bars: 50 μm. CK8, cytokeratin 8; IRS1, insulin receptor substrate 1; p-, phosphorylated; GS, glycogen synthase.

Additional experiments investigated the impact of high glucose treatment on CK8 expression and the IRS1/PI3K/ Akt/GSK3β pathway in NCTC1469 cells. As shown in Fig. (**4A**), high glucose exposure substantially reduced cellular glycogen content compared to normal glucose conditions. Notably, CK8 expression was significantly upregulated under high glucose conditions (Fig. **4B** and **C**). The treatment significantly suppressed both IRS1 expression and phosphorylation levels (Fig. **4B**, **D**, and **E**). Moreover, high glucose selectively inhibited the phosphorylation of PI3K, Akt, and GSK3β without altering their total protein levels (Fig. **4B**, **F**, **G**, and **H**). Consistent with these observations, immunohistochemical analysis revealed a dramatic decrease in GS expression (Fig. **4I** and **J**). These results suggest that high glucose-induced upregulation of CK8 and impairment of IRS1/PI3K/Akt/GSK3β signaling may collectively contribute to disrupted glycogen synthesis in NCTC1469 cells.

### 4.4 CK8 Modulates Glycogen Metabolism *via* the IRS1/PI3K/Akt/GSK3β Axis

**Fig. (5):**
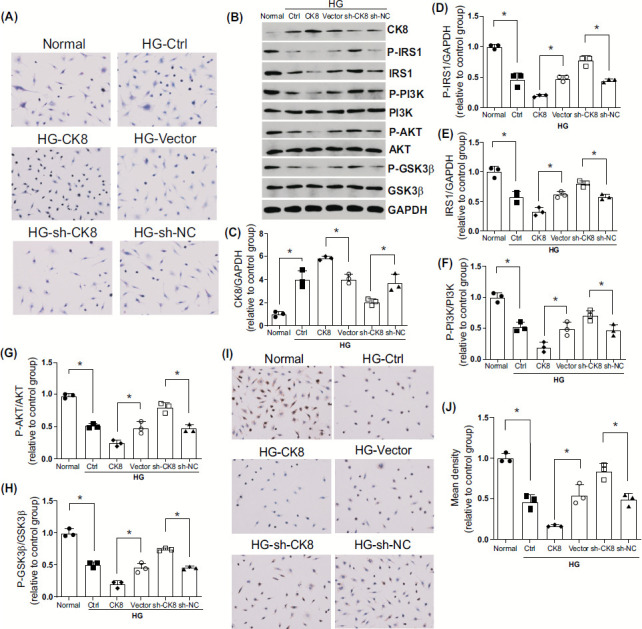
CK8 modulation in NCTC1469 cells under high glucose (33.3 mM, 24 hr). (**A**) Glycogen content was assessed in NCTC1469 cells using Periodic Acid Schiff (PAS) staining (*n*=3 independent experiments, ≥100 cells/condition). (**B**) Western blots (lysates collected 48 hr post-transfection). (**C-J**) Quantification of (**C**) CK8, (**D**) p-IRS1, (**E**) IRS1, (**F**) p-PI3K, (**G**) p-AKT, and (**H**) P-GSK3β. (**I**) Immunohistochemistry staining was performed, and (**J**) quantified to determine GS expression in NCTC1469 cells. *p<0.05. (*n*=3 biological replicates; Kruskal-Wallis with Dunn’s post-hoc). Scale bars: 50μm. Scrambled shRNA (sh-NC) served as a transfection control.

In high glucose-treated hepatocytes, CK8 overexpression further suppressed glycogen synthesis by 45% compared to controls (Fig. **5A**-**C**), whereas CK8 knockdown restored glycogen levels to near-normal (*p*<0.05). This regulation was mediated through the IRS1/PI3K/Akt/GSK3β pathway. CK8 reduced IRS1 phosphorylation by 60% (Fig. **5D** and **E**), impairing insulin signal transduction. Phosphorylation of PI3K and Akt decreased by 50% and 55%, respectively (Fig. **5F** and **G**), disrupting glucose uptake and metabolism. Reduced GSK3β phosphorylation (Ser9) by 40% (Fig. **5H**) led to sustained inhibition of GS, limiting glycogen storage (Fig. **5I** and **J**).

### 4.5 *In vivo* Validation and Broader Implications for T2DM Therapy

**Fig. (6):**
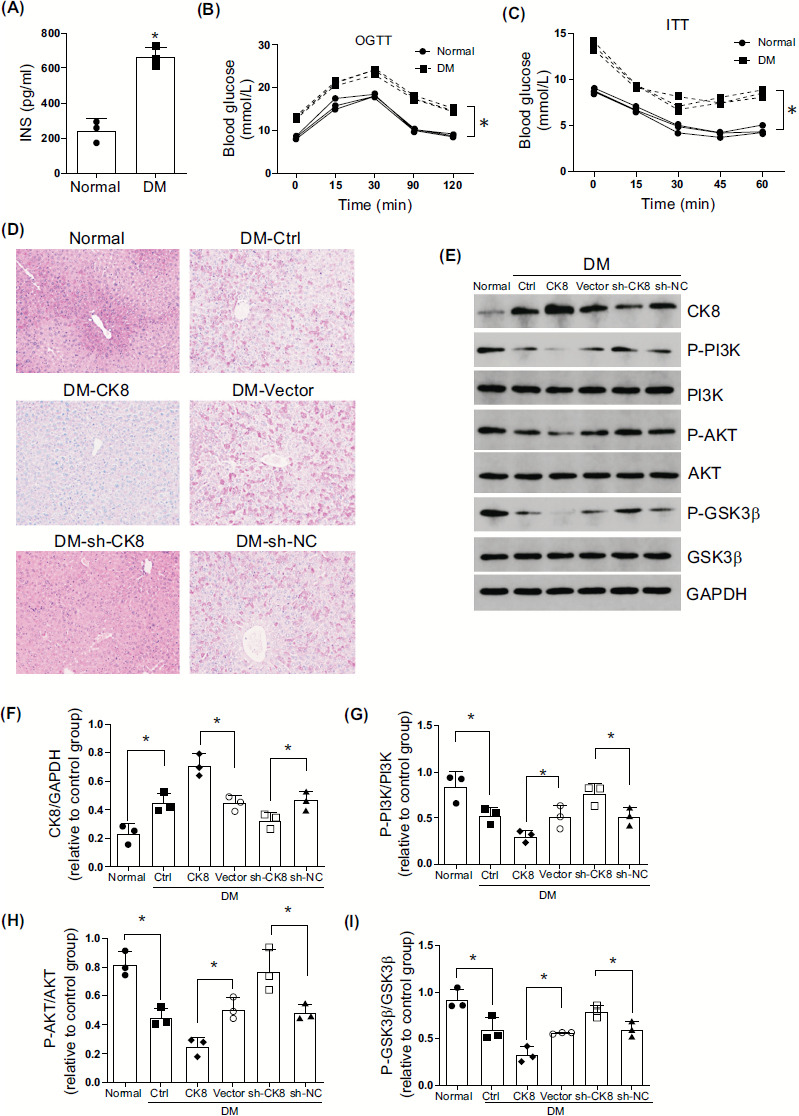
*In vivo* effects of CK8 modulation in T2D mice. T2D mice (high-fat diet + streptozotocin) received empty vector, CK8, sh-NC, or sh-CK8; controls received normal diet + buffer. (**A**) Serum insulin by ELISA (*n*=3/group). (**B**) OGTT and (**C**) ITT results. (**D**) Hepatic glycogen by PAS staining. (**E**) Representative Western blots and (F-I) quantification of (**F**) CK8, (**G**) p-PI3K, (**H**) p-Akt, and (**I**) p-GSK3β (GAPDH loading control). *p<0.05. (*n*=3/group, Kruskal-Wallis with Dunn's post-hoc). Scale bars: 50 μm. CK8, cytokeratin 8; T2D, type 2 diabetes; sh, short hairpin RNA; NC, negative control; p-, phosphorylated.

To investigate the role of CK8 in glycogen metabolism, we assessed serum insulin levels in T2D and control mice using ELISA. T2DM mice exhibited significantly elevated insulin levels compared to controls (Fig. **6A**), indicative of compensatory hyperinsulinemia. Subsequent OGTT and ITT revealed pronounced glucose metabolism dysfunction in T2DM mice, characterized by markedly higher blood glucose levels following glucose challenge (Fig. **6B**) and impaired glucose clearance after insulin administration (Fig. **6C**). In contrast, CK8 knockdown improved these parameters. PAS staining demonstrated a substantial reduction in hepatic glycogen content in T2DM mice relative to controls (Fig. **6D**), which coincided with reactivated PI3K/Akt/GSK3β signaling (Fig. **6E**-**I**). These findings highlight CK8 as a regulator of glycogen metabolism in T2DM, with dual implications. CK8 upregulation in T2DM perpetuates insulin resistance by disrupting the IRS1/PI3K/Akt/GSK3β pathway, creating a vicious cycle of hyperglycemia and glycogen depletion. Moreover, targeting CK8 could restore glycogen synthesis independently of insulin secretion, offering a novel strategy for T2DM patients with advanced β-cell dysfunction.

### 4.6 Integration with T2DM Pathophysiology

The broader significance of CK8 modulation lies in its tissue-specific effects. Unlike systemic insulin sensitizers, CK8 inhibition may selectively enhance hepatic glycogen storage without exacerbating hypoglycemia or adipose lipid accumulation. This aligns with clinical observations of glycogen-deficient livers in T2DM and suggests CK8 as a biomarker for hepatic insulin resistance.

## 5 DISCUSSION

Our study demonstrated three key findings. CK8 was significantly upregulated in T2DM liver tissues, high glucose-treated hepatocytes, and diabetic murine models, correlating with reduced hepatic glycogen content. The IRS1/PI3K/Akt/GSK3β pathway was found to be suppressed in these T2DM models. Functional studies established that CK8 negatively regulates hepatic glycogen synthesis through modulation of this pathway. These results provide novel insights into the molecular mechanisms underlying impaired glycogen storage in T2DM.

Our previous work demonstrated that CK8 expression was significantly upregulated in hepatitis C virus (HCV)-infected hepatocytes. Furthermore, forced overexpression of CK8 induced apoptosis in SMMC-7721 hepatocellular carcinoma cells [37]. The observed upregulation of CK8 across all experimental models (human, cellular, and murine) aligned with emerging evidence of cytokeratins' involvement in glucose metabolism. While traditionally recognized for their structural roles, our findings support the paradigm-shifting concept that intermediate filaments like CK8 actively participate in metabolic regulation [38]. Notably, Alam *et al.* [39] demonstrated that CK8 knockout mice exhibited improved glucose tolerance and insulin sensitivity, while Mathew *et al.* [20] reported enhanced glycogen synthesis upon CK8 downregulation in hepatocytes. Our results support these observations by mechanistically linking CK8 to the IRS1/PI3K/Akt/GSK3β pathway in the pathophysiology of T2DM.

IRS1 serves as a critical regulator of multiple cellular processes, including proliferation, apoptosis, and metabolic homeostasis [29, 40-43]. As a key component of insulin signaling pathways, IRS1 is abundantly expressed in insulin-sensitive tissues and plays a pivotal role in mediating the biological effects of insulin [44-48]. The consistent suppression of IRS1/PI3K/Akt/GSK3β signaling across our models provides compelling evidence for its central role in the glycogen synthesis impairment associated with T2DM. IRS1 downregulation in T2DM tissues corroborates its established role in insulin resistance [49-52]. Reduced PI3K/Akt phosphorylation without changes in total protein levels suggests post-translational modulation. Parallel decreases in GSK3β phosphorylation and GS expression indicate a disruption of the downstream pathway. Extensive research has established the critical involvement of the PI3K/Akt pathway in the pathogenesis of insulin resistance in T2DM [53-57]. Furthermore, IRS1 has been identified as a key upstream regulator of PI3K/Akt signaling across multiple cell types [27, 58-61]. These findings are supported by Xu *et al.* [22] and Guo *et al.* [62], who similarly implicated IRS1 and PI3K/Akt in T2DM metabolic dysregulation. In HepG2 cells, Akt has been shown to regulate glycogen metabolism *via* GSK3β and glycogen synthase (GS) [63-65]. Additionally, chloroquine treatment may enhance glucose uptake by modulating Akt-mediated GS activation in muscle cells [66-68]. Cytokeratins are primarily recognized for their role in maintaining the structural integrity of epithelial cells; however, their participation in metabolic pathways remains less well-defined [69-71]. The IRS1/PI3K/Akt/GSK3β pathway is well-established in insulin signaling and glucose metabolism, and its dysregulation is frequently linked to type 2 diabetes [72-74]. Given the critical importance of glycogen synthesis regulation in diabetes, GSK3β emerges as a key modulator of the Wnt/β-catenin signaling pathway, which governs insulin homeostasis [75]. Our study advances this knowledge by identifying CK8 as an upstream regulator of this pathway in hepatocytes.

Our multi-model data establish CK8 as a central negative regulator of hepatic glycogen synthesis through suppression of the IRS1/PI3K/Akt/GSK3β pathway. This suppression manifests as reduced phosphorylation of IRS1 (Tyr612), PI3K (Tyr458/199), Akt (Ser473), and GSK3β (Ser9), resulting in the downstream inhibition of glycogen synthase (GS) activity. These findings align with prior evidence of IRS1/PI3K/Akt dysfunction in T2DM, yet uniquely position CK8 as an upstream modulator bridging cytoskeletal dynamics to metabolic dysregulation. 

## CONCLUSION

In summary, CK8 emerges as a key regulator of hepatic glycogen synthesis, with its inhibition offering a novel, β-cell-sparing approach to T2DM management. While challenges remain in optimizing delivery and minimizing off-target effects, the hepatocyte-specificity of CK8 and its downstream positioning make it a uniquely promising target for therapeutic intervention. Future work will prioritize CK8-selective small molecules and evaluate their synergy with existing insulin sensitizers.

## STUDY LIMITATIONS

While our study has focused on the regulation of hepatic glycogen metabolism by CK8 through the IRS1/PI3K/Akt/GSK3β axis, we recognize the importance of considering its broader interactions within the cytoskeletal network and metabolic pathways. Beyond its well-characterized dimerization with CK18, emerging evidence suggests CK8 may coordinate with other intermediate filaments (*e.g.*, vimentin and desmin) to modulate insulin signaling, a possibility we are currently investigating through co-immunoprecipitation studies in primary hepatocytes. Notably, our preliminary data reveal that CK8 knockdown alters the subcellular distribution of AMPKα, suggesting potential crosstalk with the energy-sensing AMPK/mTOR (mechanistic Target of Rapamycin) pathway (unpublished observations). This is particularly relevant given recent findings that mTORC1 (mechanistic Target of Rapamycin Complex 1) inhibition can improve hepatic insulin sensitivity independent of Akt [74]. Future work will systematically map these interactions using proximity-dependent biotin identification to identify novel CK8-binding partners in diabetic hepatocytes. Such studies may reveal compensatory mechanisms employed by other cytoskeletal proteins when CK8 is inhibited, which could inform the development of combination therapies targeting multiple nodes in this regulatory network.

While our findings position CK8 as a promising therapeutic target for T2DM, we fully acknowledge the challenges in developing selective CK8 modulators given its structural and functional interdependence with other keratins, particularly CK18. The high sequence homology within the keratin family (especially in the coiled-coil dimerization domains) poses significant hurdles for achieving CK8-specific inhibition without disrupting CK18-mediated cellular functions. Our current efforts are addressing this through two parallel strategies: the structure-based drug design targeting the CK8-specific N-terminal domain (residues 1-70), where molecular modeling has identified three unique surface pockets absent in CK18; and the development of allele-specific RNAi (Ribonucleic Acid interference) using chemically modified siRNAs (small interfering RNAs) with 2'-O-methyl substitutions at positions 8-12 of the guide strand, which can achieve >90% CK8 knockdown while maintaining CK18 expression within 15% of baseline levels. Importantly, *in vivo* toxicity studies will be essential to evaluate potential off-target effects on other keratin-expressing epithelia, particularly in the intestine and pancreas, where CK8/CK18 heterodimers play a crucial role in maintaining mechanical stability. These considerations will be crucial for translating our findings into clinically viable therapies that strike a balance between metabolic efficacy and tissue safety profiles.

We acknowledged that the modest sample sizes in our human (*n*=6/group) and murine (*n*=3/group) studies may raise questions about generalizability. While these cohort sizes are consistent with comparable studies investigating rare human liver biopsies (39) and were statistically powered to detect large effect sizes (Cohen's d>1.5), we recognized the need for broader validation. To address this, we are currently establishing collaborations with multicenter biobanks to assemble a larger T2DM liver tissue cohort (targeting *n* ≥ 30/group), with enrollment projected to be completed within 36 months. Regarding *in vivo* transfection validation, while our functional outcomes (*e.g.*, 75% CK8 protein knockdown and metabolic rescue) strongly suggest successful delivery, we have now incorporated FACS (Fluorescence-Activated Cell Sorting) quantification of ZsGreen+ hepatocytes into our ongoing experiments. This standardized protocol requires only 48 hours post-injection for analysis, causing minimal delay to existing research timelines. Importantly, preliminary optimization using AAV8-GFP (Adeno-Associated Virus serotype 8-Green Fluorescent Protein) vectors has already achieved high hepatocyte transduction efficiency in pilot studies, demonstrating the technical feasibility of this approach.

## AUTHORS’ CONTRIBUTIONS

The authors confirm their contribution to the paper as follows: study conception and design: XL; data collection: JS; analysis and interpretation of results: ZW; writing, reviewing, and editing: MS. All authors reviewed the results and approved the final version of the manuscript.

## Figures and Tables

**Table 1:** Demographic and clinical characteristics of study participants.

-	T2DM	-	-	-	-		Healthy Control	-	-	-	-	-
-	1	2	3	4	5	6	1	2	3	4	5	6
Age(years)	57	60	65	65	71	64	50	45	62	60	58	61
Sex(F/M)	Female	Female	Male	Male	Male	Female	Male	Female	Female	Male	Female	Male
BMI(kg/m^2^)	23.5	24.9	26.1	25.6	24.1	25.6	25.3	22.9	23.6	23.9	22.8	22.7
HbA1c(%)	7.8%	9.2%	8.8%	7.5%	8.0%	9.4%	6.0%	5.5%	5.8%	5.2%	5.4%	5.1%
Fasting blood glucose (mmol/L)	8.2	9.4	8.8	7.6	8.3	9.6	4.9	4.6	5.0	4.5	4.6	4.3
Fasting C-peptide(ng/ml)	1.06	0.81	1.63	1.70	0.69	1.08	3.52	4.38	2.91	4.08	3.20	3.68

## Data Availability

The datasets generated and/or analyzed during this study are available from the corresponding author upon reasonable request.

## LIST OF ABBREVIATIONS

AAV8-GFP: Adeno-Associated Virus serotype 8 - Green Fluorescent Protein
Akt: Protein Kinase B
AMPK: Adenosine Monophosphate-activated protein kinase
BMI: Body Mass Index
CK8: Cytokeratin 8
DMEM: Dulbecco’s Modified Eagle Medium
ECL: Enhanced Chemiluminescence
ELISA: Enzyme-Linked Immunosorbent Assay
FACS: Fluorescence-Activated Cell Sorting
GAPDH: Glyceraldehyde-3-Phosphate Dehydrogenase
GS: Glycogen synthase
GSK3β: Glycogen Synthase Kinase 3 beta
HFD: High-fat diet
HG: High-glucose
HRP: Horseradish Peroxidase
HS: Horse Serum
IRS1: Insulin Receptor Substrate 1
ITT: Insulin Tolerance Test
mTORC1: Mechanistic Target of Rapamycin Complex 1
NCTC: National Collection of Type Cultures
OGTT: Oral Glucose Tolerance Test
PAS: Periodic Acid-Schiff
PFA: Paraformaldehyde
PI3K: Phosphoinositide 3-Kinase
PKC: Protein kinase C
RIPA: RadioImmunoPrecipitation Assay
RNAi: Ribonucleic Acid interference
RT: Room temperature
SD: Standard Deviation
SDS-PAGE: Sodium Dodecyl Sulfate-Polyacrylamide Gel Electrophoresis
siRNAs: Small interfering Ribonucleic Acids
SPSS: Statistical Package for the Social Sciences
T2DM: Type 2 Diabetes Mellitus
